# Post-mortem CT service structures in non-suspicious death investigations

**DOI:** 10.1093/bjro/tzae036

**Published:** 2024-10-29

**Authors:** Natasha Davendralingam, Amy-Lee Brookes, Mohammad Ali Shah, Susan C Shelmerdine

**Affiliations:** Department of Radiology, Tameside & Glossop NHS Foundation Trust, Ashton-under-Lynne, OL6 9RW, United Kingdom; Royal Preston Hospital, Lancashire Teaching Hospitals NHS Foundation Trust, Preston PR2 9HT, United Kingdom; Department of Radiology, St George's Hospital, London, SW17 0QT, United Kingdom; Department of Radiology, Great Ormond Street Hospital for Children NHS Foundation Trust, London, WC1N 3BH, United Kingdom; Department of Radiology, UCL Great Ormond Street Institute of Child Health, London, WC1N 3BH, United Kingdom; Department of Radiology, NIHR Great Ormond Street Hospital Biomedical Research Centre, London, WC1N 3BH, United Kingdom

**Keywords:** autopsy, postmortem, radiology, computed tomography, service models

## Abstract

Post-mortem CT (PMCT) is increasingly used in adult post-mortem investigations as a non-invasive alternative to traditional autopsies. Using PMCT supports death investigations in the face of severe pathologist workforce shortages and the less invasive nature maintains respect for cultural sensitivities. This article reviews the diverse service structures of PMCT, highlighting the importance of customizing these structures to meet the specific needs of various coronial jurisdictions. These jurisdictions often face challenges such as limited access to imaging facilities and logistical issues with geographically dispersed mortuaries. We outline options for leading and operating PMCT services, including models led by pathologists, radiologist, or a hybrid of the two; use of static, relocatable, or mobile CT scanning units; as well as making the most of existing resources such as NHS or private scanning facility scanners already in place. We also explore different PMCT reporting structures through in-house NHS radiologists, combined in-house and teleradiology, or fully outsourced teleradiology services. Each of these offerings provides different levels of efficiency, cost-effectiveness, data security and challenges to set-up. Where applicable, we present and describe real-world examples as case studies for readers interested in replicating existing models.

## Introduction

Post-mortem CT (PMCT) provides a less invasive, evidence-based adjunct (and many times alternative) to traditional invasive autopsies, supporting pathologists in the face of their workforce shortages, respecting cultural sensitivities, and improving efficiencies with death investigations.^1–11^ Digitally stored images easily facilitate second opinions, especially useful where expert availability is limited.^1^

Despite these benefits, the uptake of PMCT has been limited by access and funding issues.^1^^,^^6^^,^^8^ A common misconception is that there is only ‘one way’ to implement post-mortem (PM) imaging services and if this ‘doesn’t work’ for a particular institution, then the whole promise of PM imaging ‘won’t work either’. Understanding the range of available service structures is crucial to ensure that the most appropriate one is adopted for each jurisdiction.

The aim of this narrative review was to explore and explain the variety of possible PMCT service structures, detailing their advantages and practical considerations to guide the commissioning of effective PM imaging services. We discuss the pros and cons of the actual PM imaging scanning using static, relocatable, and mobile PMCT units, as well as existing CT scanners in NHS or private facilities. We also describe differences in PM imaging reporting structures and staffing roles, including pathologist-led, radiologist-led, and hybrid models. Where possible, we provide real-world experience and feedback on how some of these services are provided nationally, and some of the real and potential challenges of each approach.

Given the lack of literature on service structures, this narrative review not only sought publications on the topic from the last decade via peer reviewed publications (2014-2024), but we also conducted interviews and sought expert opinions from a broad range of stakeholders working in the PM imaging space, including radiographers, reporters, and coroner officers across the United Kingdom. Through these discussions and analyses, we have categorized the primary methodologies, approaches, and service frameworks in PMCT imaging.

## PM imaging scanner equipment

**Figure 1.**
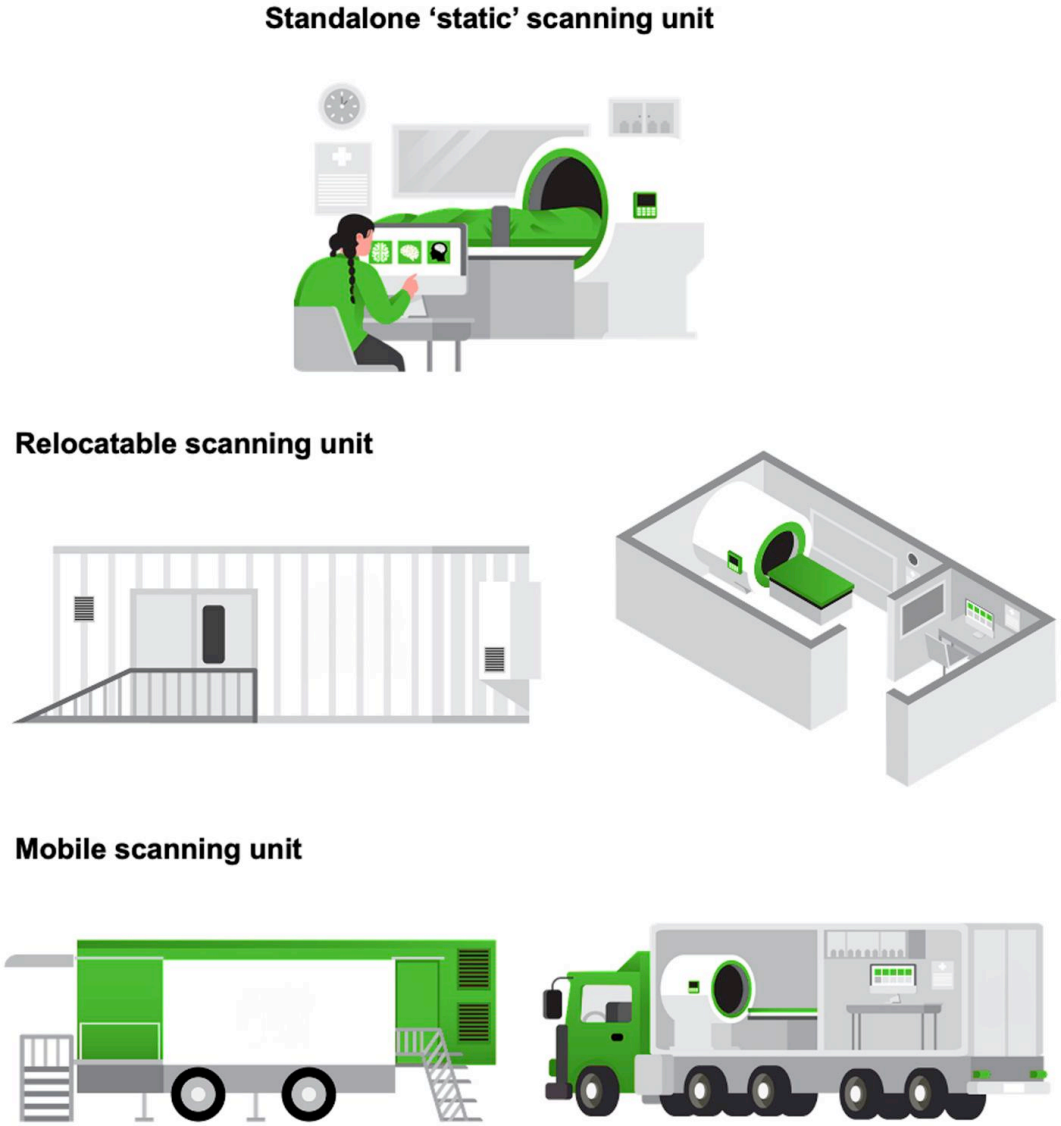
Author created diagrams of scanning unit types are detailed in Table 2 for clarity. Illustrates various PMCT scanner setups described in the text, highlighting differences in mobility and operational context.

This section discusses the types of PMCT equipment, including static, relocatable, and mobile units, and the different operational frameworks within which they function. We examine how these technologies can be integrated into current health systems and their operational implications. The distinctions among these three service setups are summarized in Table 1 and Figure 1 and elaborated further below.

**Table 1.** Advantages and disadvantages between different post mortem CT (PMCT) service setups: standalone CT, relocatable CT, and mobile CT.^11–19^

Category	Standalone CT^12^	Relocatable CT^13^^,^^14^	Mobile CT^11–13^^,^^15–19^
Advantages			
Integration	Seamless integration with hospital systems	Good integration with hospital systems	Limited integration capabilities
Comprehensive Services	Part of larger diagnostic and treatment network	Part of the hospital's service network when set up	Generally standalone service
Flexibility			
Service Reach	Fixed location	Can be moved to different locations as needed	Can travel to various locations, serving rural/underserved areas and large coronial jurisdictions or mass fatality response
Adaptability	Less adaptable to changing demands	Moderately adaptable to changing demands	Highly adaptable to changing demands
Initial Investment	Higher capital investment	Lower initial investment compared to standalone	Lowest initial investment among the three but higher transport cost between sites
Cost-effectiveness			
Resource Sharing	Dedicated to one facility (but can accept referrals from other services)	Usually dedicated to one facility (but can accept referrals from other services)	Can be shared/moved between multiple facilities
Space Requirements	Requires dedicated and permanent space, and specific building requirements and considerations (lead lined walls need to be built into building plans ahead of time)	Requires semi-permanent space, usually an outdoor parking space with access to a power source, not being used for (usually) several months at least.	Only temporary parking space needed—although the mobile unit will temporarily take up space during the day/time being used.
Disadvantages			
Costs	Higher initial and operational costs	Moderate costs, including relocation expenses	Lower initial costs but significant transport and setup costs
Flexibility	Fixed location	Moderately flexible—can be relocated if required, although usually not less than a month	Highly flexible
Utilization Rates	Needs high patient volumes to justify cost	Lower cost that standalone but still needs high patient volumes to justify cost	Can optimize utilization by moving to different locations if moving costs are mitigated by scanning volume per location
Operational Limitations	Continuous operation	Continuous operation	Limited by setup and teardown time
Environmental Constraints	Controlled environment	Generally stable environment once set up	Affected by weather and external conditions
Logistical Challenges	Not applicable	Requires logistical planning for relocation (if required)	Significant transport and storage logistics
Regulatory Compliance	Easier to maintain compliance	Complex compliance if relocation required	More complex regulatory compliance

### Dedicated static scanner/standalone unit

A standalone ‘static’ CT scanner, as opposed to a mobile solution, offers significant advantages for PM imaging. Static scanners offer superior imaging quality. They use advanced hardware and software packages that ensure high-resolution images for accurate diagnosis.^12^ This set up facilitates a higher throughput of bodies during standard operating hours, with the potential for dedicated after-hours PMCT scanning, dependent on local staffing and agreements. The system is highly efficient for continuous use in a controlled environment, which is crucial for maintaining a sustainable death investigation service that requires consistent and prompt access to imaging. However, standalone CT scanners entail higher initial and operational costs, necessitating substantial capital investment and dedicated space, including lead-lined housing. Additionally, they are less adaptable due to their fixed location, limiting flexibility in responding to changing demands or multi-site usage.^12^ These scanners are economically viable only with a high volume of cases to justify the investment and for this reason, some scanning centres without a high throughput of deceased bodies, may wish to also scan ‘live’ patients on the same unit. In summary, static CT scanners, while costly, offer high-quality imaging and efficient throughput, suitable for high-volume facilities.

### Relocatable CT unit

Relocatable CT scanners balance quality and flexibility, suitable for areas with varying case loads but require significant setup effort.^13^^,^^14^ Relocatable CT scanners demand lower capital investment costs compared to standalone units and the potential to be “shared” among multiple facilities on a rotational transport basis, however the additional cost and effort for transferring the unit from site to site means this is practically challenging if done more frequently than at 3 monthly intervals.

Modular build setups enhance the versatility of relocatable CT scanners by providing semi-permanent structures that can be quickly established, assuming the availability of appropriate electrical connections and water supply. These setups offer a stable environment comparable to fixed installations while retaining relative mobility. Staffing requirements are similar to those of standalone units, necessitating trained personnel for setup and operation at each location. However, challenges such as the need for a semi-permanent space with land lease (depending on chosen site) should be considered.

### Mobile CT unit

Mobile CT scanners offer unparalleled flexibility, capable of serving multiple locations, particularly advantageous for geographically large coronial jurisdictions with remote or difficult to access rural communities and mortuaries. This flexibility however incurs additional costs, particularly for each relocation between sites [12]. Mobile units are commonly employed in acute settings, such as in response to mass fatality incidents for disaster victim identification.^11^^,^^13^^,^^15–17^ These scanners, mounted on trucks can be easily transported to different sites, providing essential imaging capabilities in areas without fixed CT installations.

Mobile CT scanners require a smaller initial capital investment and, like relocatable units, can be shared among several facilities on a short-term basis (every few days), optimizing resources. While they may offer slightly lower image quality compared to standalone units due to hardware limitations.^18^^,^^19^ studies have demonstrated no perceptible difference in subjective image interpretation or diagnostic accuracy.^18^^,^^19^ The use of mobile CT requires advanced logistical planning and dedicated staff for transport and operation, and their availability can be affected by transportation schedules and external conditions. Consequently, mobile CT units may be valuable in geographically large jurisdictions with challenging access or as an interim solution before establishing a more permanent scanning unit.

## PM imaging service structures

In this section, we describe real-world examples of how the different scanner types described above are used in practice, including addressing data protection issues, and how these structures are adapted to suit different jurisdictions. Table 2 summarizes the differences between these service structures.

**Table 2.** Advantages and disadvantages between different post mortem CT (PMCT) service structures: pre-existing clinical CT scanners (NHS/Private), dedicated in-house/relocatable PMCT scanner and mobile PMCT scanner.^6–9^^,^^15–16^^,^^20–23^

Category	Pre-existing clinical CT scanners (NHS/Private)^6–9^^,^^20–22^	Dedicated in-house/relocatable PMCT scanner^6^^,^^8^^,^^20–23^	Mobile PMCT scanner^15^^,^^16^
Advantages			
Technology	Often available 24/7 and integrated into the hospital’s workflow.	Specifically designed and optimized for post-mortem work, including specialist staff and appropriate facilities.	Staffed by specialist personnel and can deliver similar functionality to dedicated in-house units.
Integration	Seamless integration with hospital systems.	Easier to maintain confidentiality with a secure, separate data management system.	Can maintain secure, separate systems for data management, ensuring confidentiality.
Comprehensive Services	Part of the hospital's service network.	No competition for time with clinical patients, avoiding scheduling conflicts and delays.	Flexibility and accessibility: Can be transported to various locations.
Flexibility			
Service reach	Fixed location.	Fixed location (but can be moved if required although less convenient to move compared to mobile)	Can be transported to various locations. Particularly useful in disaster response settings.
Adaptability	Less adaptable to changing demands.	Specifically designed for post-mortem cases and workflow.	Highly adaptable to changing demands.
Initial investment	Avoids substantial initial outlay for new machines.	High initial setup costs, including specialized equipment and facility construction.	Lower initial investment compared to dedicated in-house units.
Cost-effectiveness			
Resource sharing	Shared maintenance and operational costs across departments.	High operational costs, including maintenance and staffing; but can be reduced if site used by multiple coronial jurisdictions.	Theoretically can be cost-effective by sharing investment and operational costs across multiple sites. Cost can also be reduced if site used by multiple coronial jurisdictions. Cost-effective for mass disaster response.
Space requirements	Requires existing hospital space.	Requires dedicated and permanent space.	Requires semi-permanent or no permanent space, depending on the setup.
Disadvantages			
Costs	Lower initial costs but shared operational costs.	High initial and operational costs.	Lower initial costs but significant transport and setup costs.
Utilization rates	May face scheduling conflicts with clinical patients.	Utilization rate must be sufficient to justify expenses.	Can optimize utilization by moving to different locations.
Operational limitations	Competes with clinical patient scheduling and needs.	Continuous operation dedicated to post-mortem cases. Potential for shared venue with clinical cases.	Continuous operation dedicated to post-mortem cases. Potential for shared venue with clinical cases.
Logistical challenges	Requires careful logistical planning for transporting deceased bodies to avoid patient and staff distress.	Not applicable.	Transport of deceased to mobile scanner (eg, across carpark, large land space)
Confidentiality management	Requires separate data management systems to comply with the common law duty of confidentiality.	Can maintain secure, separate systems for data management, ensuring confidentiality.	Can maintain secure, separate systems for data management, ensuring confidentiality.
Operational constraints	Competes with clinical patient scheduling and needs.	Dedicated solely for post-mortem use, avoiding competition for resources.	Dedicated solely for post-mortem use, avoiding competition for resources.
Suitability for disaster response	Not suitable for rapid deployment in disaster settings.	Not portable; confined to the specific location of the facility.	Mobile CT is highly suitable for rapid deployment and providing services on-site in disaster settings.

### Use of pre-existing ‘static’ clinical CT scanners—NHS or private facilities

Using a pre-existing ‘static’ clinical CT scanner in an NHS trust or private facility offers notable advantages and disadvantages.^6–9^ This approach is cost-efficient as it leverages existing equipment, avoiding the substantial initial outlay for new machinery and sharing maintenance and operational costs across departments. Additionally, these scanners are often available 24/7, and integrated into the hospital’s workflow for emergency service work, as well as being equipped with advanced software for image pre and post-processing to ensure high-quality imaging and seamless data management.

Nevertheless, usual operational constraints such as scheduling conflicts with ‘live’ clinical patients can pose challenges. The hospital environment may also be less suited for PM cases as these require careful logistical planning for transporting bodies to avoid the distress of other staff and patients. Additionally, while General Data Protection Regulation (GDPR) rules do not apply to the deceased, the common law duty of confidentiality still does.^20–22^ To adhere to the legal requirements, data and images of the deceased should ideally be stored in a separate system to clinical scans and should be inaccessible to unauthorized personnel. Access to these images should be restricted only to users explicitly authorized by the coroner.

## Real-world applications

### Oxford Radcliffe Medicolegal Imaging Service

This centre uses a nearby NHS facility CT scanner for PM imaging out of hours (before 7 am and after 6 pm). A multidisciplinary team, including radiologists and pathologists, conducts and reviews the scans, ensuring comprehensive PM analysis.^9^

### TIC Health at the Hive, London

This private scanning facility offers dedicated PMCT and PM MRI services using a static unit. Both CT and MRI scanners are operational during specific ‘low intensity’ hours (11 am to 3 pm). This setup avoids any overlapping appointments with regular ‘live’ patient imaging schedules ensuring smooth operation and minimal disruption and dignity for all.

#### Dedicated in-house static or relocatable PMCT scanner

The primary benefit of a dedicated in-house PMCT scanner lies in its capacity to deliver immediate and efficient imaging, producing high-quality images that facilitate rapid and precise analysis. A dedicated unit is essential for addressing the imaging requirements of PM cases without competing for resources with clinical patients, thereby eliminating scheduling conflicts and minimizing delays in death investigations. Additionally, a specialized PMCT unit can be meticulously designed and optimized for PM use, including the integration of specialist staff, appropriate facilities for handling deceased bodies, and the enforcement of strict infection control protocols. Compliance with the common law duty of confidentiality is more easily managed in such a setting, as the unit can maintain a secure, segregated data management system accessible exclusively to authorized personnel.^20–22^ If the dedicated scanning unit operates within an NHS trust, there is the potential to leverage the existing NHS picture archiving and communications system (PACS). However, additional security measures should ideally be implemented to ensure PM data remains inaccessible to unauthorized individuals, such as password-protected access.

Despite these advantages, there are several drawbacks to consider. The initial setup costs for a dedicated PMCT unit can be substantial, encompassing the acquisition of specialized equipment and the construction of appropriate facilities. Ongoing operational costs, including maintenance and staffing, are also significant. Moreover, the unit's utilization rate must be sufficient to justify these expenses, which can be challenging in areas with lower volumes of PM cases. To enhance cost-effectiveness, the service can share investment and operational costs by accepting referrals from multiple jurisdictions, thereby optimizing equipment utilization and reducing potential downtime.

An alternative approach involves utilizing a relocatable CT unit instead of a static one within an in-house PMCT setup. Whether as part of a purpose-built modular configuration or as a standalone unit, this setup offers several advantages. It can be deployed and operational within a relatively short timeframe of 2 to 4 weeks, providing a robust and efficient PMCT service, particularly when positioned adjacent to an existing mortuary. Moreover, it is generally more cost-effective to establish compared to investing in a newly purchased, static, dedicated in-house PMCT scanner.

### East London Forensic Centre

This standalone mortuary and scanning facility uses an in-house static CT scanner to provide uninterrupted PM imaging services. The CT scanner is integrated into the facility's structure, ensuring a high throughput of cases and continuous operation. This set up is designed for only PM imaging, without any mixture of live and deceased patient scanning.

### Lancashire Teaching Hospitals

This service features a relocatable scanner attached to a purpose-built PM investigation facility with direct access to the on-site mortuary through a private corridor. The scanner is dedicated only to PM imaging. This setup provides flexibility in service delivery, catering to varying caseloads without the need for extensive reconfiguration or disruption to existing hospital services.^6^^,^^8^^,^^23^

#### Mobile PMCT scanner

Mobile PMCT scanners are invaluable in scenarios requiring rapid deployment, such as disaster victim identification or in remote areas where traditional imaging facilities are inaccessible. These units are designed to be highly adaptable, offering crucial imaging capabilities on-site, which can significantly expedite the identification process and aid forensic investigations. Access to an electricity source or generator is the only requirement.

### 2017 Grenfell Tower disaster

A mobile PMCT scanner played a critical role by providing on-site imaging of victims, which aided in quick identification and efficient management of the disaster aftermath.^16^

### Operation torch and operation Hounslow

Demonstrated the effectiveness of mobile scanners in complex field conditions, supporting forensic teams during mass fatality incidents and enabling prompt and accurate assessments of deceased individuals.^15^

At present, the authors are unaware of any jurisdiction using a mobile PMCT scanning unit for regular, routine PM imaging work however this would be a possibility as a stop-gap between starting a new service and establishing a more permanent solution for longer term PMCT services.

### PM imaging pathway models

**Figure 2.**
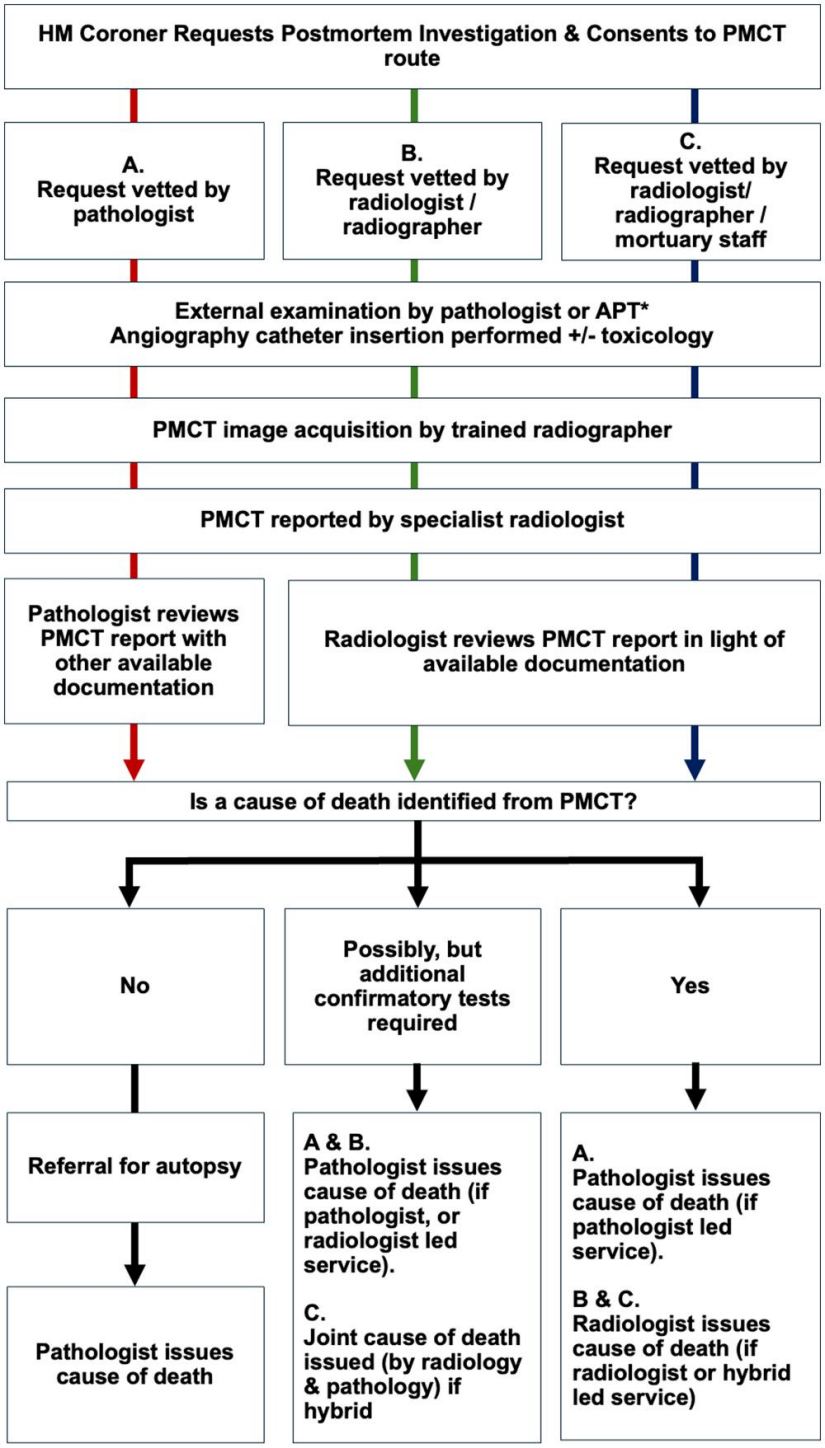
Author created illustrative pathway comparing the workflow and responsibilities of healthcare professionals under different PMCT service models: Pathway A: pathologist-led service; B: radiologist-led service; and C: hybrid model. Where a cause of death is not entirely established by the imaging a pathologist will also be required regardless of the service model. (*) It is important to note that where an external examination by the APT or pathologist raises any possibility of a non-suspicious cause of death, then a pathologist will need to be involved and a forensic autopsy may ensue.

Due to greater workforce shortages in pathology, and the fact that not all pathologists are currently trained to perform autopsies, leveraging the use of PM scanning to alleviate some of their workload is an attractive proposition. Nevertheless, it is still important to have a pathologist in the loop as PMCT is not always able to provide a cause of death nor will it always be indicated, therefore despite best efforts, an autopsy may still be required. Different models for varying levels of responsibility and organizational leadership (either via a radiologist, pathologist, or mixture of both) for a PMCT scanning service have been trialled and are described below. Similarities and differences between these models are outlined in Figure 2.

#### Pathologist-led model

The 2021 national guidance from the Royal College of Radiologists (RCR) and the Royal College of Pathologists supports a pathologist-led PMCT service model.^1^ In this framework, the pathologist maintains a pivotal role, assessing the suitability of cases referred by the coroner for PM examination. After reviewing the PMCT report, prepared by a radiologist with appropriate training and considering all relevant documentation including the external body examination, the pathologist is responsible for issuing the final cause of death. The pathologist ultimately decides whether the PMCT findings are sufficient to determine the cause of death or if an invasive autopsy is required. This model is practised in Leicester Royal Infirmary, East London Forensic Centre (ELF) and ORAMIS (Oxford).^8^^,^^9^^,^^23^

At the Leicester Royal Infirmary, a pathologist evaluates each coroner referred case to assess whether PMCT has the potential to help establish the cause of death. Mortuary technicians prepare the body for the imaging unit, and the scan is performed according to a specific protocol which may include whole-body imaging and where necessary, additional targeted coronary angiography. In this facility, coronary angiography is performed for cases when the Agatston coronary calcium score is less than 400. On-site local/in-house radiologists trained in PMCT review and report the scans. The pathologist then determines whether the PMCT findings are sufficient to establish a cause of death or if an invasive autopsy is required. This decision is communicated to the Coroner, who then informs the family.^8^

At ELF, cases are referred to and vetted by a suitably trained radiologist, rather than a pathologist, to evaluate suitability for scanning and potential for establishing a cause of death. The radiologist will also advise on suitability for coronary angiography based on pre-defined criteria and demographics, as well as patient’s medical history. External examination is conducted by a pathologist prior to CT scanning. The scan is then performed and reported remotely by a radiologist. The pathologist issues the final cause of death based on the PMCT report findings alongside all other available evidence.^23^

In this service, the exclusion criteria for PMCT suitability and angiography suitability are detailed in Tables 3 and 4, respectively. The exclusion criteria developed have been adapted from the PM cross-sectional imaging in adults for non-forensic deaths guidelines from the RCR, the forensic and PM radiography guidance of the Society of Radiographers and practices of other PM imaging centers including Leicester Royal Infirmary and Lancashire Teaching Hospitals NHS Trust.^1^^,^^6^^,^^8^^,^^21^

**Table 3.** PMCT exclusion criteria.

PMCT exclusion criteria
Complex hospital deaths
Bariatric deceased exceeding the CT table weight or gantry bore limit
Anatomical features which prohibit movement through the gantry (eg, contraction of skeletal framework with deceased in awkward positions, deceased too wide)
Cases needing histology (eg, known asbestosis)
Epilepsy as a sole factor in the peri mortem history
Metabolic pathology resulting from a known dysregulation occurring because of a diagnosed condition (eg, ketoacidosis in diabetes mellitus)
Presence of a penetrating injury with the item still protruding from the deceased’s body which impedes the bodies travel through the bore of the scanner (the scan may have to wait until after the item is removed)
Presence of firearms or explosives. If known prior to scanning these must be removed by trained personnel before the body can be scanned. If detected during CT, the deceased cannot be further scanned or moved until the weapon has been removed by trained personnel.
Deceased soaked in an accelerant (petrol, etc.) or anything that produces fumes.
Deceased embedded in another material which causes the individual to exceed the weight or gantry bore limit of the scanner (eg, concrete, mud, sealed in a barrel of oil
Any case specifically marked as not suitable by His Majesty’s coroner

The exclusion criteria developed have been adapted from the post-mortem cross-sectional imaging in adults for non-forensic deaths guidelines from the Royal College of Pathologists, the forensic and post-mortem radiography guidance of the Society of Radiographers and practices of other post mortem imaging centers including Leicester Royal Infirmary and Lancashire Teaching Hospitals NHS Trust.^1^^,^^6^^,^^8^^,^^21^

**Table 4.** PMCT angiography exclusion criteria.

PMCT angiography exclusion criteria
Decomposition
Hangings
Severe Trauma (person under train, decapitation etc.)
Intravenous drug user
Level 4 Infections
Fire deaths
Paediatric cases
Coronary calcium score >400

A level 4 infection is caused by any microbe that poses a high risk of aerosol-transmitted infection (eg, Biosafety Level 4, BSL-4). These may include but are not limited to infections such as dangerous viral or prion infections such as Ebola, Lassa fever, Bolivian haemorrhagic fever etc. The exclusion criteria developed have been adapted from the post-mortem cross-sectional imaging in adults for non-forensic deaths guidelines from the Royal College of Pathologists, the forensic and post-mortem radiography guidance of the Society of Radiographers and practices of other post mortem imaging centers including Leicester Royal Infirmary and Lancashire Teaching Hospitals NHS Trust.^1^^,^^6^^,^^8^^,^^21^

#### Radiologist-led model

In this model, the radiologist is the central role to the PMCT scanning service. They are responsible for vetting suitable cases for imaging (referred by the coroner), which can also be performed by suitably trained radiographers. Radiologists issue the final cause of death after evaluation of available documentation (eg, coroner case information summary, medical summary and relevant hospital or GP letters), and results from the external examination. However, if the radiologist is unable to issue a cause of death, the case is then referred to a pathologist to continue the death investigation. The pathologist will have access to the radiologist’s PMCT report for consideration. This model is practised at Lancashire Teaching Hospitals.^6^

The PMCT service at the Lancashire Teaching Hospitals NHS Trust (LTH) is a collaboration between Lancashire County Council and LTH working in conjunction with a third party provider, who supply the modular unit and CT scanner. Another third party provider provides RIS, PACS and body transportation of the deceased to LTH. This collaboration has allowed PMCT to be provided at a similar cost to their prior invasive autopsy service to the community with over 1500 cases scanned each year.^6^^,^^24^ The modular unit, which contains the CT scanner and a control room, is attached to the existing LTH mortuary by a purpose-built corridor which also houses a modular cold room. The service itself is NHS led with full-time trained PMCT radiographers undertaking the scans which are reported by in-house NHS radiologists as a part of their job plan.

The role of the pathologist has been replaced by Anatomical Pathology Technicians (APT) to undertake the external examination for non-suspicious cases. This introduction of an APT led external examination service evolved due to a dire lack of available pathologists within this jurisdiction, rather than any malintent to exclude pathologists from the service structure.^6^

#### Hybrid model

The hybrid model involves a shared leadership and responsibility between radiologists and pathologists working collaboratively to deliver a PMCT service. On receipt of a PM examination request from the coroner, the case is referred to the radiologist for vetting and determination of suitability for imaging. Trained radiographers or mortuary staff may also vet the referrals, but radiologists remain available for advice and retain overall responsibility. Once vetted, the need for an angiographic study will be considered at the discretion of the vetting radiologist based on the case circumstances and any pre-established exclusion criteria.

If PMCT is deemed unsuitable, the case is referred directly for invasive autopsy. Pathologists or trained APTs perform external examinations of the bodies (with quality ensured by annual pathologist-led checks). If appropriate, APTs will also insert the angiography catheter at this stage.

A standard imaging protocol is used for PMCT image acquisition with angiography and calcium scoring performed as appropriate. Once acquired, images are sent to a reporting radiologist along with the external examination map, all PM documents and any relevant previous imaging. On review of the images, the radiologist will issue the cause of death and will send the report directly to the coroner. In some instances, a cause of death is found but further specialist input or investigation is needed (eg, toxicological assessment, tissue biopsy or a more detailed external examination). In this circumstance, findings are forwarded to the pathologist for review and the cause of death subsequently issued to the coroner through joint discussion between radiologists and pathologist. Where a cause of death is not identified, the case is referred to the pathologist for autopsy. This model promotes an integrated nature of delivery between pathology, radiology, and mortuary services.

The hybrid model is currently employed in South Manchester with a PMCT service provided by Tameside and Glossop NHS trust.^25^

The key differences between the service models are summarized in Table 5.

**Table 5.** Key differences in PMCT imaging pathway models.

Category	Pathologist-led model	Radiologist-led model	Hybrid model
Leadership role	Pathologist leads the process	Radiologist leads the process	Shared responsibility between radiologists and pathologists
**Case vetting**	Pathologist assesses suitability for PMCT	Radiologist (or trained radiographer) vets suitability	Radiologist or trained staff vets; radiologists retain responsibility
**Cause of death**	Issued by the pathologist	Issued by the radiologist	Issued collaboratively by radiologist and pathologist in complex cases
**External examination**	Performed by a pathologist	Performed by Anatomical Pathology Technicians (APT)	Performed by pathologist or trained APT with annual quality checks by a pathologist
**Examples of practice**	Leicester Royal Infirmary, East London Forensic Centre, ORAMIS	Lancashire Teaching Hospitals NHS Trust	South Manchester (Tameside and Glossop NHS Trust)

### PM imaging reporting structures

In all service model structures, a suitably trained radiologist would report the PMCT imaging however how these radiologists are employed and where they are geographically based may be different depending on local staffing and expertise. The advantages and disadvantages between different PMCT reporting structures are summarized in Table 5.

#### In-house reporting

##### Advantages

In-house reporting employs radiologists local to the scanning site (who are usually otherwise occupied reporting ‘live’ patients) to provide PM imaging reports. Depending on workload and job plans, there is the potential for real-time interpretations and prompt turnaround times. This approach ensures convenient communication between on-site radiologists and pathologists, fostering enhanced collaboration that leads to more accurate and consistent findings. Moreover, it offers opportunities for upskilling local staff, promoting professional development and potentially improving job satisfaction within the facility.^6–9^

##### Disadvantages

This method involves variable costs, which may be higher due to the work often being performed outside of regular job plans or hours and reliant on the local staff's willingness to undertake additional work and training. Furthermore, the availability of trained personnel may be subject to change depending on clinical service pressures. The use of a shared PACS platform (ie, NHS cases with PM imaging cases) could introduce data security risks, necessitating stringent measures to protect sensitive information. Whilst local radiologists may be comfortable with reporting PMCT, they may be less willing or able to take time from work to attend coronial inquests where their presence is required.^20–22^

#### In-house reporting with Ad-Hoc teleradiology

##### Advantages

Combining in-house reporting with PM specialized ad-hoc teleradiology support provides access to expert advice, which is particularly beneficial for complex situations. This hybrid model is cost-efficient for covering staff absences or obtaining second opinions when necessary, maintaining the advantages of in-house reporting such as timely and accurate interpretations and fostering confidence among local staff.

##### Disadvantages

The model may introduce delays due to the time required for obtaining external opinions, and conflicts may arise between in-house and external teleradiologists over diagnostic recommendations, possibly necessitating multidisciplinary discussions to resolve discrepancies. Additionally, the dependence on ad-hoc support might lead to higher per-unit costs for occasional imaging studies and foster a reliance on external opinions, potentially diminishing local expertise over time.^26^^,^^27^

#### Private teleradiology (outsourced)

##### Advantages

Outsourcing to private PM specialized teleradiology services offers access to a broad range of accredited radiologists with dedicated training in this area, enhancing the quality of PM analysis and providing a scalable solution for handling a varying caseload. This model may lead to cost savings by reducing the need for an in-house team and the associated training and internal audit of reporting quality. With pre-agreed turnaround times, it ensures reliable and efficient management of backlogs.

##### Disadvantages

There is the chance however this could complicate the coordination with local staff and introduce variability in service quality due to the diversity of reporting staff. Depending on the provider and the volume of cases referred, outsourcing could incur higher costs, especially for urgent cases requiring immediate attention. Additionally, remote radiologists might find it inconvenient to travel long distances for in-person testimony at coroner's inquests, though strategic assignments of reporters can mitigate these issues and many coroners are now amendable to virtual testimony.^26^^,^^27^

**Figure 3.**
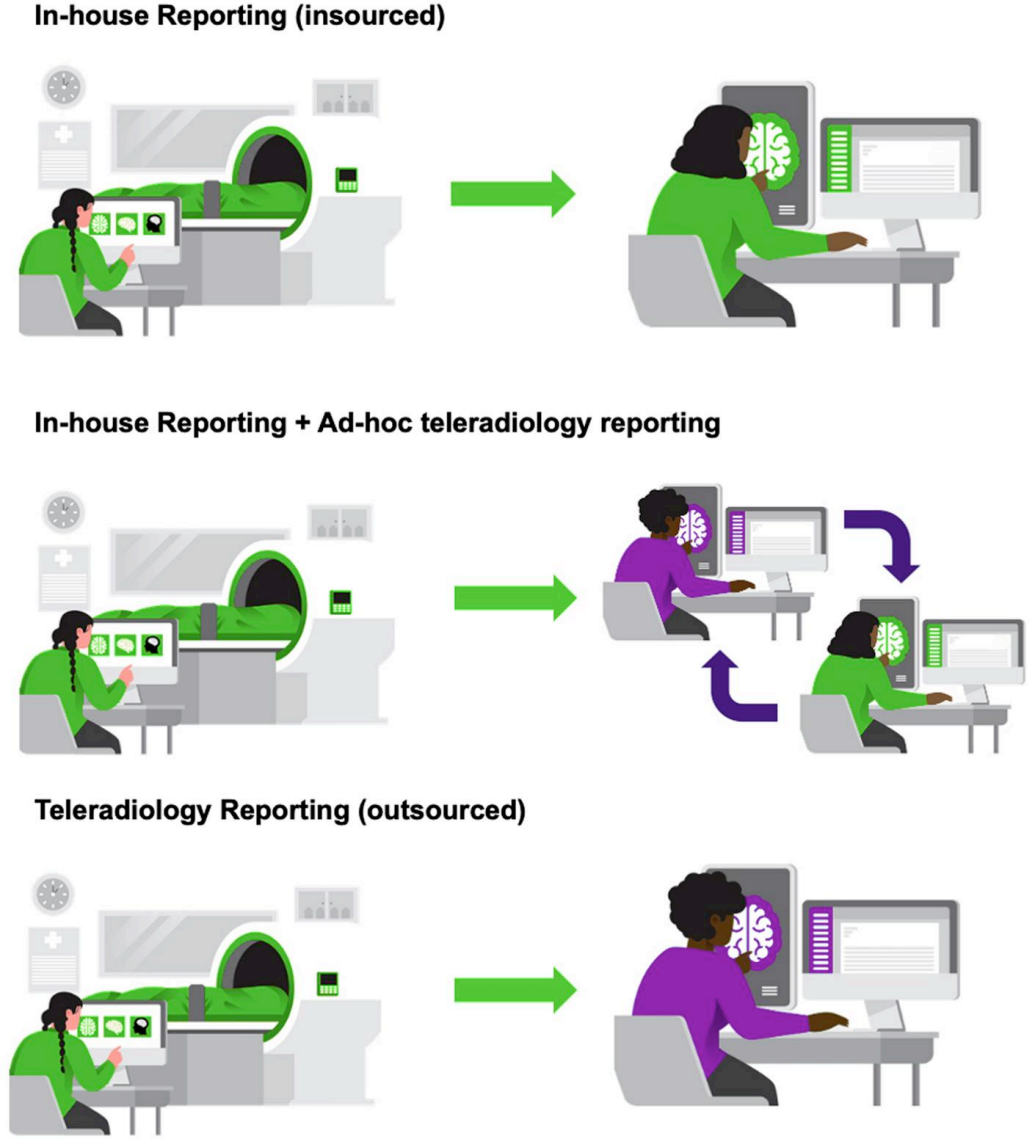
Author created diagrammatic representation of different PMCT reporting structures ranging from fully in-house to fully outsourced setups.

A diagrammatic representation of different radiologist reporting structures is illustrated in Figure 3.

## Conclusion

Post-mortem CT offers a valuable, less invasive alternative to traditional autopsies, adaptable to the specific needs of diverse coronial jurisdictions. In this article we have highlighted how different PMCT service models can be adapted and used to suit local needs, from static scanners to mobile units and also the different leadership structures using pathologists, radiologists or a hybrid of both specialities to issue the final cause of death.

Successful implementation of PMCT services requires alignment with local needs and addressing challenges related to access, funding, and data security. Tailoring PMCT services to local needs can overcome challenges related to access, funding, and data security, making it both cost-effective and efficient.

## Data Availability

Data sharing is not applicable to this article as no datasets were generated. Summary of findings from previously published articles are provided already in figures within this review.
